# Are Community Gardening and Horticultural Interventions Beneficial for Psychosocial Well-Being? A Meta-Analysis

**DOI:** 10.3390/ijerph17103584

**Published:** 2020-05-20

**Authors:** Giuseppina Spano, Marina D’Este, Vincenzo Giannico, Giuseppe Carrus, Mario Elia, Raffaele Lafortezza, Angelo Panno, Giovanni Sanesi

**Affiliations:** 1 Department of Agricultural and Environmental Sciences, University of Bari Aldo Moro, 70126 Bari, Italy; marina.deste@uniba.it (M.D.); vincenzo.giannico@uniba.it (V.G.); mario.elia@uniba.it (M.E.); raffaele.lafortezza@uniba.it (R.L.); giovanni.sanesi@uniba.it (G.S.); 2 Department of Education, Roma Tre University, 00185 Rome, Italy; giuseppe.carrus@uniroma3.it; 3 Department of Geography, The University of Hong Kong, Centennial Campus, Hong Kong, China; 4 Department of Human Science, European University of Rome, 00163 Rome, Italy; angelo.panno@unier.it

**Keywords:** meta-analysis, horticulture, well-being, social support, neighborhood cohesion, human health–environment interaction, psychosocial health

## Abstract

Recent literature has revealed the positive effect of gardening on human health; however, empirical evidence on the effects of gardening-based programs on psychosocial well-being is scant. This meta-analysis aims to examine the scientific literature on the effect of community gardening or horticultural interventions on a variety of outcomes related to psychosocial well-being, such as social cohesion, networking, social support, and trust. From 383 bibliographic records retrieved (from 1975 to 2019), seven studies with a total of 22 effect sizes were selected on the basis of the Preferred Reporting Items for Systematic Reviews and Meta-Analyses (PRISMA) guidelines. Meta-analytic findings on 11 comparisons indicate a positive and moderate effect of horticultural or gardening interventions on psychosocial well-being. Moderation analysis shows a greater effect size in individualistic than collectivistic cultures. A greater effect size was also observed in studies involving community gardening compared to horticultural intervention. Nevertheless, an effect of publication bias and study heterogeneity has been detected. Despite the presence of a large number of qualitative studies on the effect of horticulture/gardening on psychosocial well-being, quantitative studies are lacking. There is a strong need to advance into further high-quality studies on this research topic given that gardening has promising applied implications for human health, the community, and sustainable city management.

## 1. Introduction

The interaction between people and nature in urban areas has considerable impacts on environmental and life quality, as well as public health. Nature in cities provides essential services enhancing biodiversity and promoting a restorative environment for a healthy lifestyle, as well as recreational opportunities for socializing among citizens [1,2,3,4,5,6]. The latter can be considered an emerging trend of studies in the field; therefore, we have decided to focus on it as the outcome of our research.

The response of human beings to nature finds an emerging application in urban agriculture and community gardening for therapeutic purposes [7]. The two activities are often considered equivalent and the terms used as synonyms but in fact, the official definitions consider community gardening as a specific subgroup of horticultural activity. According to the definition of the American Horticultural Therapy Association (AHTA), horticultural therapy represents a systematic treatment intervention that uses natural features, plant care, and gardening activities to increase the physical and psychological health of participants. Horticultural therapy can involve a wide number of activities, such as indoor and outdoor gardening, passive interaction with nature, collection of natural elements and cooking, whereas community gardening can be defined as a particular category of horticultural activities that takes place in a public environment, regardless of the context, and that involves a group of people who are commonly involved in gardening activities [8]. In the framework of nature-based therapy, people are exposed to treatments that include observation, the manipulation of plants and flowers, and the creation of food products (e.g., jams) as a path for therapeutic purposes, relearning abilities, and building a learning environment. The clinical practice accumulated through these types of treatment and rehabilitation interventions has resulted in the development of good practices, clinical protocols, and associations, such as the AHTA, which legislatively regulates what has become a well-recognized clinical profession [9]. Gardening has proved to exert a positive impact on several health outcomes. The activities related to plant care, both indoor and outdoor, have been proved to be associated with the significant improvement of physical and mental health and interpersonal relationships [10,11]. There are countless horticultural interventions involving people belonging to cognitively, physically or socially vulnerable populations who experience behavioral, emotional, and social interaction difficulties [12]; such people are the elderly [13,14], patients with Alzheimer’s disease or dementia [15,16], patients with psychiatric diseases, such as schizophrenia [17], with learning disabilities [18], injured brain [19], or refugees [20]. A recent meta-analysis [21] on the effect of gardening on health has collected 22 case studies with pre-post experimental designs and control groups. Meta-analytic results showed a significant positive effect of gardening on a large number of physical (e.g., body mass index, physical activity level) and psychological health outcomes (e.g., mood, well-being, life satisfaction, and cognitive functioning) as well as an increase in sense of community and life satisfaction. Overall, the literature provides evidence on the positive effect of gardening on physical and mental health. Lack of evidence, however, can be seen concerning the effects of community gardening and horticultural therapy on the social dimensions of psychological well-being of gardeners, i.e., the psychosocial well-being, which can be defined as the perception of the individual on the quality of his/her relationships in his/her community, in the neighborhood and in any social group to which he/she belongs [22]. Examples are: social inclusion and occupational participation [23], neighborhood connection [24], social cohesion [25], social connectedness [26], and social interaction and participation [27]. Nevertheless, the impact of horticultural therapy on social experience in terms of increased social exchanges and participation has not been well documented through experimental designs yet [28]. In this regard, it is fair to underline that most of the published studies on psychosocial well-being are qualitative and use data collection through focus groups and non-systematic interviews. Qualitative measures are not often accompanied by experimental designs manipulating the independent variable (e.g., gardeners vs. non-gardeners), which would enable a demonstration of the treatment’s effectiveness. As far as we know, no quantitative synthesis, (i.e., meta-analysis) of the benefits of horticultural and gardening therapy on psychosocial well-being outcomes has been published to date. Meta-analysis is a quantitative and comprehensive evaluation of the results of several studies; its primary role is to synthetically address the discrepancies between the findings of different studies on the same topic, or the contingencies of the studies themselves [29].

The overall aim of this study is to carry out a meta-analysis to investigate the beneficial effects of community gardening and horticultural interventions on psychosocial well-being (i.e., neighborhood cohesion and attachment; perceived neighborhood aesthetics; closeness; trust; value uniformity; social cohesion; social support; emotional/informational, tangible, and affectionate social support; positive social interaction; social involvement; collective efficacy; a sense of community; perception with membership, influence, meeting needs, and with a shared emotional connection; loneliness; and social networking). Specific aims concern investigating whether there is: (a) evidence of improvement in psychosocial well-being outcomes after community gardening or horticultural treatment (pre-post); (b) evidence of improvement in terms of psychosocial well-being in those who have benefitted from community gardening or horticultural treatment (gardeners) compared to those who have not benefitted from it (non-gardeners); (c) the presence of cross-cultural differences of the benefits mentioned, i.e., individualistic (Western) vs. collectivistic (Eastern) cultures; and (d) a difference concerning the most commonly used treatments (i.e., community gardening and horticultural therapy).

## 2. Materials and Methods

### 2.1. Data Collection

The procedure for selecting the publications to be included in the meta-analysis followed the Preferred Reporting Items for Systematic Reviews and Meta-Analyses (PRISMA) statement [30]. The objective of the bibliographic research was to identify studies focusing on community gardening and horticultural interventions in relation to psychosocial well-being. Only sample studies with experimental manipulation (i.e., experimental and control groups) were included. The bibliographic search through Scopus was concluded in October 2019. After several preliminary tests, the final query was composed of the following keywords:

“urban agriculture” OR “community garden” OR “gardening” OR “allotment” OR “allotment garden” OR “horticulture” OR “horticultural therapy”.

And “inclusion” OR “inclusiveness” OR “social integration” OR “social interaction” OR “social cohesion” OR “social health” OR “social support” OR “social wellbeing” OR “social well-being” OR “psychosocial” OR “social participation” OR “social involvement” OR “social engagement”.

The eligible publications were obtained by accessing the library of the University of Bari. The Scopus database resulted in 383 records from 1975 to 2019. The inclusion criteria for our meta-analysis were as follows: (a) English or Italian language, (b) articles, (c) quantitative study, (d) use of urban green space or peri-urban, and (e) sample study. Qualitative studies, case studies, those conducted in extra-urban green environments, and studies in which there was a lack of environmental information, despite requesting data from the corresponding authors, were excluded. The research articles were screened by language and document type and, subsequently, by title, abstract, and keywords to establish their eligibility.

### 2.2. Data Extraction

**Figure 1:**
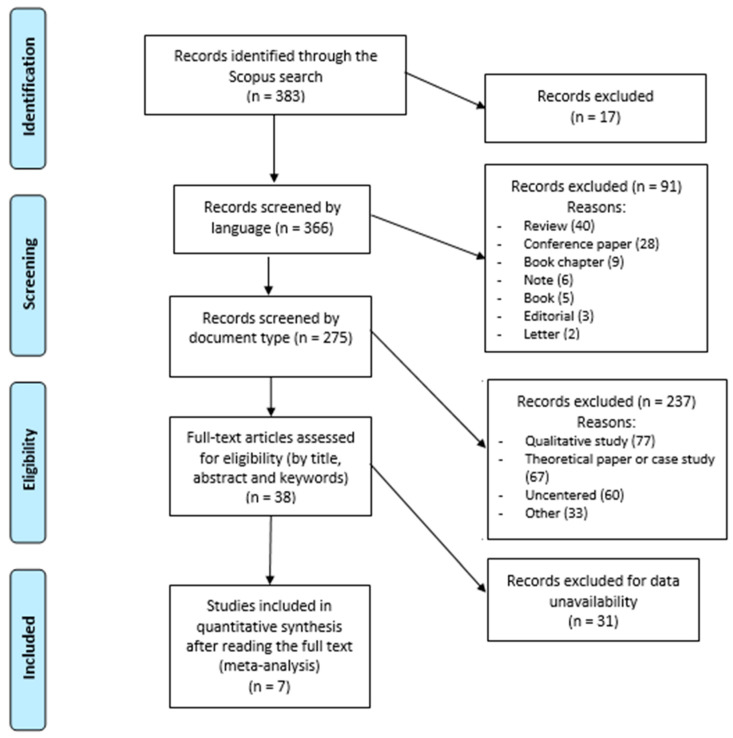
Systematic review flowchart detailing the literature search, number of abstracts screened, and full texts retrieved.

After the screening procedure, we included seven documents (Table 1) in our analysis. The PRISMA diagram shows the detailed study selection process (see Figure 1). For each indicator considered, in three of the seven studies [31,32,33] we collected the sample sizes, means, and standard deviations (SDs) of the intervention follow-up for both the control group and experimental group. The remaining four studies [20,34,35,36] presented a comparison only between baseline and follow-up of the experimental group, for a total of 11 indicators considered. One study [35] provided comparisons among three groups, i.e., home gardeners, community gardeners, and non-gardeners. In the analysis, we considered only the latter two groups because the features of the first group did not meet our inclusion criteria. A total of 22 effect sizes are discussed.

### 2.3. Statistical Analysis

Meta-analysis was performed using a “meta” package [37] and R statistical environment (Version 3.3.2; R Core Team, Vienna, Austria). We classified the case studies (Table 2) into six subgroups: (I) studies conducted in the US or UK, (II) studies conducted in the Asian countries, (III) studies that applied horticultural intervention, (IV) studies that applied intervention based on community gardening, (V) studies that provided pre- and post-intervention scores (baseline and follow-up), and (VI) studies involving at least one experimental group or gardeners and a control group or non-gardeners. Based on this classification, the following comparisons were investigated (Table 2): (a) pre-post (before vs. after treatment); (b) participant type (experimental group or gardeners vs. control group or non-gardeners); (c) country/city (studies performed in the US and UK vs. Asian countries or city), and (d) treatment type (community gardening vs. horticultural intervention). Some of the case studies provided incomplete information because only the mean and SD of the follow-up scores were presented. Therefore, the number of studies changes according to the comparison considered. For each comparison, the mean, SD and 95% Confidence Interval (CI) range of the effect size were calculated by applying the random effect model, which assumes that selected studies are different due to intrinsic factors of the studies included in the meta-analysis (i.e., in case of significant heterogeneity). Heterogeneity between and within the subgroups was analyzed by means of the Q test. Cochran’s Q is used as a test of heterogeneity among effect sizes. A significant Q_w_ value suggests significant heterogeneity within a group, while a significant Q_b_ suggests significant differences between groups.

The metric used for calculating the effect size of the comparisons between subgroups was Hedges’ *g* standardized mean difference [38]. Lastly, a forest plot was produced for displaying the value of the effect size and the relative 95% CI for each case study included in the meta-analysis.

Furthermore, we investigated the presence of publication bias among the examined case studies. Publication bias is the tendency to avoid publishing a study with non-significant results. This could lead to an overestimation of the effectiveness of a treatment or therapy and, consequently, a distortion in the results of the meta-analysis [39]. To verify the presence of publication bias, the Egger’s test was conducted. In the event that the Egger’s test reveals the presence of publication bias, the fail-safe *N* calculation using the Rosenthal approach [40] and trim and fill analysis are necessary. The purpose of these analyses is to identify how many studies (or comparisons) were missing to reach an adequate number for a meta-analysis free from publication bias [41].

## 3. Results

### 3.1. Descriptive Characteristics of Included Studies

Table 1 presents the seven studies included in the analysis. Each study was divided with respect to the wide range of psychosocial well-being outcomes examined and measures administered. Two studies [20,36] share the same outcome, i.e., social support. Studies on cross-cultural differences came from the US (2 studies; 9 outcomes), Hong Kong, China (2 studies; 6 outcomes), and Japan (2 studies; 6 outcomes). Only one of the studies included in this meta-analysis has been carried out in Europe (i.e., UK; 1 outcome); it was merged with the group from the US according to country/city, as both share an individualistic culture compared to the collectivistic culture, which is typical of Asian countries. Gardening types include gardening articulated in the community (outdoor group activities), allotment (each plot-holders’ garden is an individual allotment plot but in close proximity to other plots), and indoor gardening (carried out in the multiple function room of the nursing homes with plants of each participant placed along the window side of the room) (5 studies; 13 outcomes), and horticultural intervention, which unlike gardening, was not necessarily a group activity (2 studies; 9 outcomes). The sample size was highly variable among the studies, from a minimum of 45 to a maximum of 978 participants. The participants were mostly adults or elderly and ranged in average age from about 45 to more than 80 years, with a variable percentage of men and women, except for an all-women study sample. The sample groups were generally community dwellers, but there were also specific samples such as cancer patients, refugees, and residents in low-rent housing estates. The measures used were also very variable, that is, some of them included specific validated scales for measuring a given outcome, e.g., [31], others used subscales to measure different aspects of psychosocial well-being, e.g., [20], and still others only a few items [35]. A higher score denoted greater psychosocial well-being for the participants, with the exception of the “Revised University of California, Los Angeles (UCLA) Loneliness Scale” [42] for which a higher score indicated higher perceived loneliness and, therefore, lower psychosocial well-being. To perform statistical analyses, the score on this scale was reversed.

### 3.2. Meta-Analysis Results and Publication Bias

**Figure 2:**
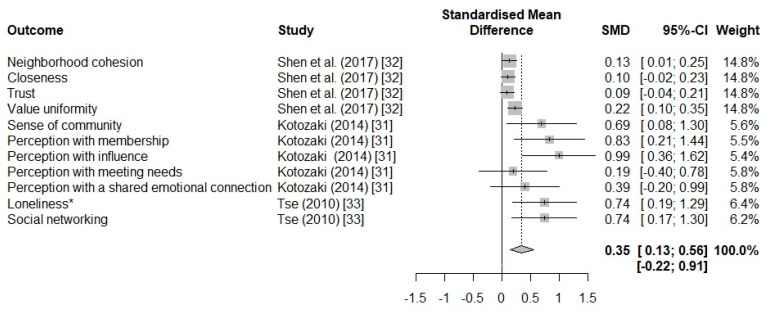
Forest plot showing the results of the meta-analysis on the effect sizes, including the 95% confidence interval effect size, of gardening and horticulture on social health outcomes for 11 comparisons. (* The scores of the “Revised University of California, Los Angeles (UCLA) Loneliness Scale” [42] were reversed, since a higher score indicated greater perceived loneliness, thus lower psychosocial well-being). SMD = Standardized Mean Difference; CI = Confidence Interval.

**Figure 3:**
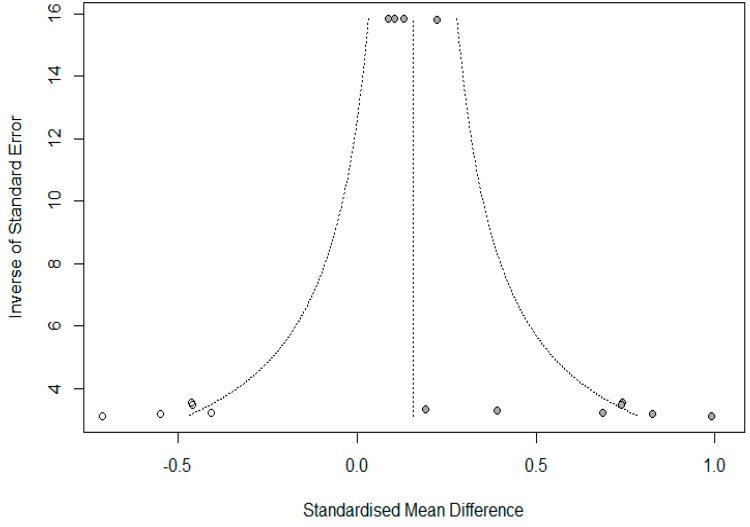
A funnel plot to assess potential publication bias. Measures of effect size (standardized mean differences) are represented on the *x*-axis and for study precision (the inverse of standard error) on the *y*-axis. The gray and white circles represent observed data (11 comparisons) and added data (5 studies), respectively.

The results of the meta-analysis are shown in Figure 2. Only three studies presented mean and SD for both the control and experimental groups; therefore, only 11 comparisons are presented in the forest plot on the overall effect of gardening. All studies show a positive effect after an intervention based on gardening or horticulture. As shown in Figure 2, community gardening or horticultural interventions have an overall significant positive effect on the psychosocial well-being of practitioners (Hedges’ *g* = 0.35, 95% CI: 0.13–0.56). The 95% CI of the overall pooled effect size did not overlap zero, thus showing a significant effect. Conversely, a confidence interval containing the “zero” value implies that there is no certain or significant treatment effect, and therefore the treatment can have a positive, negative, or no impact [43]. According to Cohen’s [44] guidelines, the effect size of the relationship between community gardening or horticultural interventions and psychosocial well-being was moderate. By dividing the available data into subgroups, it was possible to analyze the heterogeneity within and between the subgroups (Table 2). The 95% CI of effect size did not overlap zero for studies carried out in the US or UK, for those who conducted a gardening intervention, for the overall comparison between pre- and post-intervention and for studies with gardeners as an experimental group and non-gardeners as a control group (Table 2). The effect size between the subgroups was significantly different for both country/city (Q = 9.35, *p* = 0.01) and treatment (Q = 7.50, *p* = 0.01). In a comparison by country/city, studies conducted in the US or UK showed a higher effect size on average, while comparing by treatment community gardening showed an overall higher effect size than horticultural therapy. Within-study heterogeneity was found for all the subgroups, indicating evidence of heterogeneity of variance among the effect sizes, except for the one concerning horticultural therapy (Q = 13.40, *p* = 0.0987). However, where the meta-analysis has a small number of studies, as in our case, the power of this test is low and represents more of an indication than evidence [45]. To investigate the presence of publication bias, Egger’s test was performed (t = 4.79, df = 9, *p* < 0.001). The test was significant and indicated the presence of publication bias, therefore, fail-safe N calculation and trim and fill analysis was needed. Fail-safe N calculations demonstrated that 115 additional studies with effect size zero could be added to the meta-analysis before the result lost statistical significance, while trim and fill analysis showed that five comparisons (white dots in Figure 3) were missing from our collection of studies due to publication bias. Adding those five missing comparisons to the original dataset, the adjusted effect size of the overall effect of community gardening and horticultural intervention decreased (mean = 0.16; 95% CI: −0.12–0.43) suggesting that the effect of publication bias was relevant.

## 4. Discussion

This is the first attempt to perform a meta-analysis on the psychosocial well-being of community gardening and horticultural interventions. Previous meta-analyses focused on the effect of gardening on a wide range of health outcomes, such as depression, stress, mood, as well as body mass index and obesity as physical health measures [21]. Our meta-analysis investigated the effect of treatment or therapy involving gardening activities on outcomes related to psychosocial well-being (e.g., neighborhood cohesion, trust, and social networking). Our results of seven case studies suggested an overall positive effect of this kind of horticultural or gardening intervention on psychosocial well-being. Quantitative studies with available data were predominantly conducted in the US, Hong Kong, and Japan, with only one in Europe (i.e., the UK). Treatment settings were community gardening activities or horticultural therapy sessions. The wide variability of participants, settings, and measuring instruments is likely the reason for heterogeneity among the selected studies, as evidenced by our findings and in line with previous evidence on the beneficial effect of gardening on health [21]. Indeed, subgroup analysis showed a significant between-group difference, where positive influence was higher in studies conducted in individualistic cultures (US and UK) and in those involving community gardening users. This finding is consistent with literature which highlights the positive influence of community gardening as an opportunity to spread social interaction, mutual support, and cohesion. A potential mechanism underlying this relationship might be the relational context of such activities [25,27,46], in particular, in individualistic cultures characterized by a lesser habit of being involved in co-participation activities and freer emotional expression [47]. The studies examined greatly varied in sample and treatment characteristics. In some cases, the studies followed a standardized procedure that provided for a wide-ranging recruitment phase for city or neighborhood residents, followed by predefined sessions that included, for example, walks and field activities [32]. Other studies recruited gardeners directly in urban allotments or in a specific area of the city, without intervention, and therefore in the absence of a baseline measurement [34,35], or special samples such as refugees and women living in disaster areas [31]. Finally, it is worth mentioning two studies that explored the possible benefits of gardening activities for promoting healthy aging [33,36]. Hawkins et al. [36] reported a significant difference in the decrease in perceived stress, but no difference in terms of social support. The authors hypothesized that social interaction promoted by group activity does not necessarily translate into perceived social support. Social support probably needs more time to be established, as it depends on care and the feeling of belonging with respect to social interaction, which is *hic et nunc* [48]. Tse [33] found a significant decrease in the level of perceived loneliness in a group of elderly residents in nursing homes. Unfortunately, this result is not very generalizable as the gardening activity was indoor.

The use of this type of therapy for the prevention and treatment of age-related disorders is growing. A large number of studies report the benefits of gardening activities on the cognitive, physical, and psychosocial well-being of elderly users [49]. Additionally, they have long term effects when integrated and implemented in daily living. For instance, community or home gardening has shown to affect a large number of positive habits in the elderly, such as physical activity, eating vegetables, connection with the neighborhood, and a reason to live [24]. Gardening seems to trigger a number of important protective factors for active and healthy aging. Nevertheless, more comparison studies between gardeners and non-gardeners are needed to provide strong empirical evidence [13].

Even though we found a significant moderate effect size in the relationship between practitioners of gardening and horticultural interventions, the findings of our meta-analysis must be taken with caution since an effect of publication bias was detected. Publication bias is a limitation that is often found in meta-analyses, especially when so few studies are available, as is this case [39]. Moreover, the studies included in our meta-analysis showed a high level of heterogeneity in terms of study samples, outcomes, experimental sessions, settings, and measures used, which are sometimes limited to a few questionnaire items. Thus, a greater number of studies are needed to investigate whether and how the effect size varies as a function of these moderators. Nonetheless, the scientific interest in the field is rising and deserves a greater amount of attention. Despite these limitations, our meta-analysis takes on a double meaning. First of all, it demonstrates the existence of a quantifiable positive effect of horticultural activities on the psychosociological health of gardeners. But more importantly, it underlines the need to combine the great amount of qualitative evidence with as many quantitative studies, especially with groups of individuals who lack awareness of their well-being and have difficulty in recognizing and verbalizing subjective improvements in well-being, such as people with psychotic disorders, Alzheimer’s Disease, learning disabilities, and an injured brain [15,17,18,19]. Thus, our results might be considered as a first attempt to support a new promising avenue of research with relevant applied implications. Future research should synthesize these results using clearly defined terminology, a systematic treatment protocol, and objective and quantitative measurements and outcomes, which are recommended for qualitative, explorative, and descriptive studies [50].

## 5. Conclusions

Despite a large number of systematic reviews on the effect of horticultural interventions in terms of community gardening and horticultural therapy on psychosocial well-being, to the best of our knowledge, no meta-analyses have been performed so far. Since gardening and horticultural interventions might give rise to several physical and psychological benefits, with relevant outcomes for public health, more experimental studies investigating a causal relationship are warranted. Although our results must be taken with caution, they would seem to suggest a further applied implication of these interventions (i.e., psychosocial functioning aimed to improve citizens’ quality of life and sense of community). Thus, to provide useful therapeutic interventions with strong scientific value and clinical significance, a corpus of studies with well-defined protocols would need to be developed. Based on these observations, a research agenda providing guidelines aimed to design gardening- and horticultural-based interventions may be a relevant tool to foster analyses in the field. It is worth advancing into further high-quality studies given that gardening has promising applied implications for human health, the community, and sustainable city management.

## Figures and Tables

**Table 1:** Characteristics of experimental comparisons included in the meta-analysis. EG = Experimental Group; CG = Control Group. In the allotment gardening intervention, each plot-holders garden deals individually with an allotment plot but in close proximity to one another. In the indoor gardening intervention, all gardening activities were carried out in a room of the nursing homes.

Study	Country/City	Intervention	Outcome	Number of Participants		Measure
				EG	CG	
Shen et al. (2017) [32]	Hong Kong	Horticultural intervention	Neighborhood cohesion	502	476	Neighborhood cohesion scale (NCS)
	Hong Kong	Horticultural intervention	Closeness	502	476	NCS II item
	Hong Kong	Horticultural intervention	Trust	502	476	NCS III item
	Hong Kong	Horticultural intervention	Value uniformity	502	476	NCS IV item
Soga et al. (2017) [34]	Japan	Allotment gardening	Social cohesion	165	167	Social Cohesion and Trust Scale
Gerber et al. (2017) [20]	US	Community gardening	Social support	22	28	Medical Outcomes Study Social Support Survey (MOS SSS)
	US	Community gardening	Emotional/informational social support	22	28	MOS SSS sub-scale
	US	Community gardening	Tangible social support	22	28	MOS SSS sub-scale
	US	Community gardening	Affectionate support	22	28	MOS SSS sub-scale
	US	Community gardening	Positive social interaction	22	28	MOS SSS sub-scale
Litt et al. (2015) [35]	US	Community gardening	Perceived neighborhood aesthetics	63	130	6 items
	US	Community gardening	Social Involvement	63	131	4 items
	US	Community gardening	Collective efficacy	62	120	12 items
	US	Community gardening	Neighborhood attachment	62	131	6 items
Kotozaki (2014) [31]	Japan	Horticultural intervention	Sense of community	22	23	Sense of Community Index 2 (SCI-2)
	Japan	Horticultural intervention	Perception with membership	22	23	SCI-2 membership
	Japan	Horticultural intervention	Perception with influence	22	23	SCI-2 influence
	Japan	Horticultural intervention	Perception with meeting needs	22	23	SCI-2 meeting needs
	Japan	Horticultural intervention	Perception with a shared emotional connection	22	23	SCI-2 shared emotional connection
Hawkins et al. (2011) [36]	UK	Allotment gardening	Social support	25	23	Social provisions scale
Tse (2010) [33]	Hong Kong	Indoor gardening	Loneliness	26	27	Revised UCLA Loneliness Scale
	Hong Kong	Indoor gardening	Social networking	26	27	Lubben Social Network Scale

**Table 2:** Meta-analysis results of each subgroup considered. NOTE: Cochran’s Q is used as a test of heterogeneity among effect sizes. A significant Q_w_ value suggests significant heterogeneity within a group, while a significant Q_b_ suggests significant differences between groups. EG = Experimental Group; *n.s.* = non-significant; SE = Standard Error; CI = Confidence Interval.

Subgroups	No. of Comparisons	Effect Size			Heterogeneity	Between-Subgroup Difference
		Mean	SE	95% CI		
**Country/City**						
US + UK	10	0.73	0.202	0.48–0.97	Q_W_ (df = 9) = 24.00; *p* < 0.01	Q_b_ (df = 1) = 9.35; *p* < 0.01
Asia	12	0.15	0.510	−0.19–0.49	Q_W_ (df = 11) = 52.11; *p* < 0.0001	
**Treatment**						
Horticultural intervention	9	0.05	0.150	−0.12–0.22	Q_W_ (df = 8) = 13.40; *n.s.*	Q_b_ (df = 1) = 7.50; *p* < 0.01
Community gardening	13	0.55	0.566	0.19–0.92	Q_W_ (df = 12) = 77.44; *p* < 0.0001	
**Before/after (only EG)**						
Pre/post	11	0.29	0.412	0.01–0.59	Q_W_ (df = 10) = 31.84; *p* < 0.001	_
**Participant**						
Gardeners/non-gardeners	22	0.39	0.466	0.16–0.62	Q_W_ (df = 21) = 187.71; *p* < 0.0001	_
